# Enhanced Dual-Axis Rotation Modulation Scheme for Inertial Navigation Systems Using a 64-Position Approach

**DOI:** 10.3390/s26061796

**Published:** 2026-03-12

**Authors:** Hongmei Chen, Zhaoyang Wang, Han Sun, Dongbing Gu, Cunxiao Miao, Wen Ye

**Affiliations:** 1 Department of Electrical Engineering, Henan University of Technology, Zhengzhou 450001, China; chenhongmei@haut.edu.cn (H.C.); 1460492086@stu.haut.edu.cn (Z.W.); 2 Guizhou Tuzhi Information Technology Co., Ltd., Guiyang 550001, China; 3 Department of Computer Science and Electronic Engineering, University of Essex, Colchester CO4 3SQ, UK; 4 Department of Mechanical Engineering, University of Science and Technology Beijing, Beijing 100083, China; miaocx@ustb.edu.cn; 5 National Institute of Metrology of China, Beijing 100029, China

**Keywords:** inertial navigation system, rotation modulation scheme, dual axis rotation, error compensation

## Abstract

Rotational modulation improves strapdown inertial navigation system (SINS) by periodically reorienting the inertial measurement unit (IMU) to convert slowly varying sensor errors into manageable, cancelable components. However, existing dual-axis schemes may accumulate large total rotation angles and introduce delayed error balancing, which results in non-negligible residual attitude errors and degrades real-time navigation accuracy. To overcome these limitations, we propose an odd-symmetric dual-axis rotation strategy that jointly optimizes the rotation order and dwell positions to maximize error cancellation on each axis and across axes while constraining cumulative rotation. Based on this principle, we design a 64-position rotation scheme and derive its IMU error modulation/suppression characteristics, including gyroscope drift, accelerometer bias, scale-factor errors, and misalignment (installation) errors, and we quantify their effects on attitude and velocity. Simulations show that the proposed scheme reduces position and velocity errors by more than 60% compared to a 16-position scheme, and decreases longitude error, east-velocity error, and yaw error by more than 30% relative to a 32-position scheme. Experiments further validate consistent improvements in position, velocity, and attitude accuracy, demonstrating the effectiveness of the proposed rotational design for dual-axis SINS.

## 1. Introduction

The inertial navigation system (INS) is recognized for its ability to provide comprehensive navigation parameters, stable continuous output and passive signal concealment; therefore, it has shown unique advantages in many applications [1]. However, a significant drawback of INS is the accumulation of navigation errors over time [2]. Among various forms of INS, the strap-down inertial navigation system (SINS) stands out as a fully autonomous navigation solution that does not depend on external information [3]. Its exceptional performance in positioning and attitude measurement has led to widespread use in fields such as aviation, marine and vehicle navigation [4]. However, the navigation accuracy of SINS depends to a large extent on the performance of inertial sensors [5]. To address the decline in navigation accuracy caused by sensor errors, rotation modulation technology has emerged as an effective method for eradicating errors. By periodically rotating the IMU, it can significantly mitigate the impact of constant and installation errors. It also provides state calibration, error compensation and initial alignment, thereby enhancing the long-term stability of the navigation system. The application of rotation modulation technology in INS represents a significant area of research. Its core idea involves modulating the IMU errors of inertial sensors into high-frequency signals through rotational motion, enabling self-compensation of these errors. Depending on the number of rotating axes, rotation modulation can be categorized into single-axis, dual-axis and tri-axis schemes.

Fiber optic gyroscopes (FOGs) [6,7,8] have become particularly popular in rotation modulation technology due to their simpler production process compared to laser gyroscopes, as well as their enhanced stability and accuracy. For instance, high-precision FOG produced by Boeing utilizes rotation modulation technology [9]. Research has demonstrated the promising potential of rotation modulation technology, revealing that it can significantly enhance the performance of FOG within SINS [10,11,12]. This indicates the importance of further exploring rotation modulation to improve navigation accuracy and system reliability. Single-axis rotation modulation is a technique designed to minimize attitude errors through rotational motion. Simulation results indicate that under ideal conditions, it can significantly improve the measurement accuracy of horizontal gravity vector components [13]. Huang et al. [14] present a single-axis rotation modulation method that enhances inertial sensor performance by minimizing attitude errors and converting bias into high-frequency signals. Simulations demonstrate that this approach significantly improves the measurement accuracy of the horizontal gravity vector component under ideal conditions. Notably, reciprocating rotation has been found to yield higher accuracy compared to single-axis rotation modulation. Wang et al. [15] introduce a lateral SINS self-compensation method based on single-axis rotation modulation. The results indicate that their method effectively reduces IMU errors, thereby enhancing the accuracy of polar long-duration navigation. Additionally, Ralston et al. [16] outline six guidelines for rotation modulation, offering valuable insights for optimizing systems that incorporate self-calibration and rotation modulation functions.

Recent advancements in INS have led to the development of various rotation modulation techniques aimed at enhancing accuracy and compensating for output errors in IMU. For example, Weng et al. [17] introduced a single-axis bidirectional rotation stop system in conjunction with a dual-axis rotation system. Although this dual-axis approach may introduce fluctuations in position errors, the overall navigation accuracy can be significantly improved. To enhance initial alignment accuracy, a 6-position alignment scheme was reported in [18]. Expanding upon this foundation, Zheng et al. [19] introduced an 8-position dual-axis rotation self-calibration method and showed that it can address a wider range of errors, including those related to gravity. Experimental results demonstrate that their 8-position scheme achieves over a 50% improvement in accuracy compared to the traditional 6-position scheme, with the most significant enhancement observed in position accuracy. Yuan et al. [20] utilized error propagation equations to analyze the traditional 8-position rotation scheme in INS, proposing an enhanced 8-position scheme and a novel 16-position rotation strategy. The latter aims to address issues such as inertial sensor drift, scale factors and alignment errors, although it still requires experimental validation for practical applications. Li et al. [21] combined a single-axis continuous rotation scheme with a dual-axis multi-position transformation to develop a 12-position dual-axis rotation scheme. Similarly, Jing et al. [22] proposed a new 12-position rotation scheme for the semi-strapdown inertial navigation system (SSINS) based on micro-electromechanical system (MEMS). This scheme effectively mitigates residual errors caused by modulation angular rate errors, leading to enhanced position and attitude accuracy by over 56%. However, the combined effects of their scheme on SSINS navigation and the motor angular rate error model have not been thoroughly evaluated, which raises concerns about the potential for inaccurate estimations. Additionally, Seo et al. [23] proposed a calibration method that leverages gyroscope drift to offset errors as the position of the IMU changes, thereby enhancing the navigation performance of ring laser gyroscope-based RINS. This method implements a dual-axis 16-position rotation scheme for system-level indirect calibration and analyzes the effects of IMU attitude changes on gyroscope drift. Experimental validation indicates that this approach can significantly improve the long-term navigation accuracy of RINS. However, its applicability is limited, as it only supports system-level calibration for z-axis upward and downward positions. In SSINS, the 12-position and 16-position rotation schemes have increasingly recognized the importance of error modeling and system calibration. However, they exhibit shortcomings in terms of generality and dynamic validation.

Zha et al. [24] introduced an enhanced comprehensive error compensation scheme that significantly improved the position accuracy of the 16-position dual-axis rotation inertial navigation system (RINS). A 16-position dual-axis rotation scheme was proposed in [25] for redundant IMU, achieving significantly improved enhancements. To address increased oscillation frequencies of attitude errors caused by rotation modulation, He et al. [26] proposed a 16-position dual-axis rotation strategy that effectively improved horizontal axis attitude error accuracy. Wei et al. [27] presented a 16-position dual-axis diagonal rotation IMU solution, which addressed relative error issues in axial modulation inertial sensors, achieving reductions in longitude and latitude errors, though lacking experimental verification. While the 16-position dual-axis rotation scheme effectively compensates for IMU errors and improves position and attitude accuracy, it faces challenges during rotation modulation. Excessive cumulative rotation angles and delays in error compensation reduce its effectiveness and limit engineering applications. Therefore, further research into rotation schemes with more positions is necessary.

Several studies have explored advanced rotation schemes to enhance inertial navigation accuracy. Sun et al. [28] proposed a 32-position dual-axis rotation scheme that splits each 180° rotation into two 90° segments, pausing at each position to compensate for second harmonic errors. Building on this, Li et al. [29] introduced a 32-position scheme with experimental results showing a 29.8% improvement over the traditional 16-position scheme. Similarly, Wei et al. [30] presented a 32-position RINS rotation scheme that significantly reduced the impact of IMU errors. To achieve further enhancements, Fan et al. [31] proposed a unified 48-position rotation scheme integrating self-calibration and rotation modulation. Despite these advances, these schemes have had limited success in reducing attitude errors. While rotation schemes with 32 or more positions improve error compensation and are effective in second harmonic suppression and redundant IMU integration, they often lead to excessive cumulative rotation angles and delayed error compensation. Consequently, most are simple extensions of the 16-position scheme and do not effectively address issues of excessive accumulated angles and timely compensation.

Advancements in rotation modulation technology have elevated tri-axis rotation modulation as a highly accurate method for enhancing INS. To mitigate navigation errors caused by the coupling between gyroscope outputs and Earth’s rotation, Guan et al. [32] introduced the tri-axis rotation inertial navigation system (TRINS), designed in the inertial coordinate system. Zhang et al. [33] further analyzed the impact of yaw motion on TRINS operating on a static base, result a reduction in maximum radial position error by 0.6 nautical miles over 12 h, though other error metrics showed limited improvement. Addressing limitations in the rotation excitation sequence, Hu et al. [34] proposed an orthogonal transformation combined with a virtual Kalman filter platform based on TRINS self-calibration, significantly enhancing the scale factors and installation accuracy of gyroscopes and accelerometers. Lu et al. [35] advanced this by developing a TRINS scheme utilizing FOG, enabling continuous rotation of the IMU along three axes. This design reduced errors, mitigated velocity oscillations, and improved short-term navigation accuracy. However, issues like inaccurate rack installations and encoder misalignments continued to affect attitude outputs.To address these issues, Liu et al. [36] investigated the impact of installation errors and proposed a self-calibration method based on recursive least squares (RLSs). Additionally, Lu et al. [37] presented an improved TRINS rotation modulation scheme that integrates a multi-position stop strategy with continuous rotation. Their results showed a 50% improvement in navigation accuracy in simulations and 25% in real-world tests, marking significant progress for FOG-based RINS.These studies demonstrate that tri-axis rotation schemes enhance system accuracy and robustness in challenging environments. Nonetheless, the increased structural complexity of TRINS, with performance gains similar to dual-axis systems, suggests that dual-axis rotation systems may offer better practical value. Dual-axis systems balance complexity and performance, making them more suitable for applications that prioritize simplicity and reliability. The symmetrical properties of functions are essential for eliminating errors [38,39]. The rotation modulation scheme is designed to fully utilize the error propagation characteristics associated with these odd symmetrical properties.

To address this issue, this paper proposes a 64-position rotation scheme to enhance the error modulation capability of inertial devices with the following main contributions.

This new scheme addresses issues such as excessive cumulative angles of the rotating axis during rotation modulation and the inability of current rotation schemes to compensate for IMU errors promptly. We propose an optimal rotation scheme that incorporates odd symmetry in the rotation sequence to maximize benefits on each axis and across axes, thereby minimizing error propagation.

To evaluate the effectiveness of the proposed rotation scheme, the paper conducts a detailed calculation and analysis of the propagation laws of IMU errors, scale factor errors and installation errors. Additionally, the study examines how these errors affect residual attitude and velocity errors generated during the rotation modulation process.

The paper further substantiates the proposed 64-position rotation scheme by conducting simulations and experimental analyses of various rotation schemes. The results demonstrate that the new scheme significantly outperforms in terms of attitude and velocity errors, confirming its higher accuracy and reliability.

This paper is structured as follows. Section 2 introduces the theory of rotation modulation and analyzes the maximum cumulative angle of the traditional scheme. Section 3 presents the 64-position rotation scheme and investigates its maximum cumulative angle. Section 4 focuses on the analysis of the attitude errors across three dual-axis rotation schemes. Section 5 examines velocity errors in the dual-axis rotation schemes. Section 6 carries out the simulation analysis of the five schemes. Section 7 conducts experimental verification. Section 8 summarizes the paper with a summary of the research and its implications.

## 2. Preliminary

### 2.1. Rotation Modulation Theory

**Figure 1:**
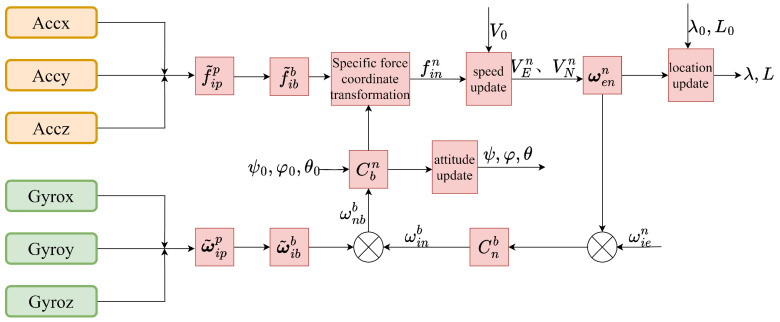
Principle of rotating SINS algorithm.

The fiber optic SINS integrates a rotating mechanism with an angle measuring device, which is mounted on the outer frame of the FOG system. Utilizing the SINS algorithm, this system performs navigation computations effectively. The attitude of the IMU is determined through a recursive algorithm, which subsequently provides essential attitude information [40]. The primary block diagram illustrating the rotation modulation is presented in Figure 1. The IMU consists of three optical gyroscopes and three flexible quartz accelerometers, along with a dual-axis indexing rotation mechanism. This device can rotate along two orthogonal axes and features both position-locking and rate-rotation capabilities. It is mounted on the rotating mechanism, establishing a novel rotation coordinate system in conjunction with the mechanism [41,42]. Here, the symbol *b* system denotes the carrier coordinate, *p* system signifies the rotating coordinate, *i* system represents the inertial coordinate, and *n* system denotes the navigation coordinate, the *p* system and *b* system coincide at the initial instant.

According to the error equation of the SINS and the algorithm principle of the RINS, the error propagation equation of the RINS is derived [43]:

(1) $$\dot{\phi }=-\omega_{in}^{n}\times \phi +\delta \omega_{in}^{n}-C_{b}^{n}C_{p}^{b}\delta \omega_{ip}^{p}$$

(2) $$\begin{array}{cc} \delta \dot{v} & =f^{n}\times \phi +C_{b}^{n}C_{p}^{b}\delta f_{ip}^{p}-\left(2\omega_{ie}^{n}+\omega_{en}^{n}\right)\times \delta v\\ \; & \;-\left(2\delta \omega_{ie}^{n}+\delta \omega_{en}^{n}\right)\times v-\delta g \end{array}$$

(3) $$\delta \dot{p}=\delta v$$

where $\phi ={\left[\begin{array}{ccc} \phi_{E} & \phi_{N} & \phi_{U} \end{array}\right]}^{T}$ is the attitude angle error, ω and δω are the angular velocity and error respectively, *f* and δf are the specific force and error respectively, *v* and δv are the velocity and error respectively, δg is the gravity deviation, and $C_{1}^{2}$ is the transformation matrix from coordinate system 1 to system 2. In rotating SINS, the inertial element measures the value of the *p* system relative to the *i* system, with its signal output as followed:

(4) $${\tilde{\omega }}_{ip}^{p}=\left(I+S_{g}\right)\left[I+{\left(\Delta C_{g}^{p}\right)}^{T}\right]{\hat{\omega }}_{ip}^{p}+\varepsilon +\eta_{g}$$

(5) $${\tilde{f}}_{ip}^{p}=\left(I+S_{a}\right)\left[I+{\left(\Delta C_{a}^{p}\right)}^{T}\right]{\hat{f}}_{ip}^{p}+\nabla +\eta_{a}$$

where $S_{g}$ and $S_{a}$ are the scale factor errors of the gyro and accelerometer, respectively. $\Delta C_{g}^{p}$ and $\Delta C_{a}^{p}$ are the installation errors of the gyro and accelerometer, respectively. ${\hat{\omega }}_{ip}^{p}$ is the actual angular velocity input of the gyro, and ${\hat{f}}_{ip}^{p}$ is the actual specific force input of the accelerometer. ε and ∇ are the gyro drift and accelerometer bias, respectively. $\eta_{g}$ and $\eta_{a}$ are the gyro and accelerometer random noise, respectively. From (4) and (5), the output error of the inertial device is:

(6) $$\delta \omega_{ip}^{p}=\left[S_{g}+{\left(\Delta C_{g}^{p}\right)}^{T}\right]{\hat{\omega }}_{ip}^{p}+\varepsilon +\eta_{g}$$

(7) $$\delta f_{ip}^{p}=\left[S_{a}+{\left(\Delta C_{a}^{p}\right)}^{T}\right]{\hat{f}}_{ip}^{p}+\nabla +\eta_{a}$$

The gyroscope and accelerometer random errors are simplified as white noise for analytical tractability and to highlight the impact of different rotation sequences.

After rotation modulation, the output error is:

(8) $$\begin{array}{cc} C_{p}^{b}\left(t\right)\delta \omega_{ip}^{p} & =C_{p}^{b}\left\{\left[S_{g}+{\left(\Delta C_{g}^{p}\right)}^{T}\right]{\hat{\omega }}_{ip}^{p}+\varepsilon +\eta_{g}\right\}\\ \; & =C_{p}^{b}S_{g}{\hat{\omega }}_{ip}^{p}+C_{p}^{b}{\left(\Delta C_{g}^{p}\right)}^{T}{\hat{\omega }}_{ip}^{p}+C_{p}^{b}\left(\varepsilon +\eta_{g}\right) \end{array}$$

(9) $$\begin{array}{cc} C_{p}^{b}\left(t\right)\delta f_{ip}^{p} & =C_{p}^{b}\left\{\left[S_{a}+{\left(\Delta C_{a}^{p}\right)}^{T}\right]{\hat{\;f}}_{ip}^{p}+\nabla +\eta_{a}\right\}\\ \; & =C_{p}^{b}S_{a}{\hat{\;f}}_{ip}^{p}+C_{p}^{b}{\left(\Delta C_{a}^{p}\right)}^{T}{\hat{\;f}}_{ip}^{p}+C_{p}^{b}\left(\nabla +\eta_{a}\right) \end{array}$$

where $C_{p}^{b}$ is the transformation matrix from *p* to *b*, specifically:

(10) $$\begin{array}{cc} C_{p}^{b} & =\left[\begin{array}{ccc} \cos\beta & 0 & \sin\beta \\ 0 & 1 & 0\\ -\sin\beta & 0 & \cos\beta \end{array}\right]\left[\begin{array}{ccc} \cos\alpha & -\sin\alpha & 0\\ \sin\alpha & \cos\alpha & 0\\ 0 & 0 & 1 \end{array}\right]\\ \; & =\left[\begin{array}{ccc} \cos\alpha \cos\beta & -\sin\alpha \cos\beta & \sin\beta \\ \sin\alpha & \cos\alpha & 0\\ -\cos\alpha \sin\beta & \sin\alpha \sin\beta & \cos\beta \end{array}\right] \end{array}$$

where $\alpha =\omega_{z}*t$ and $\beta =\omega_{y}*t$ is the rotation angle of the *z* axis and *y* axis, ω is the angular velocity, the rotation angles of the two axes change alternately in sequence.

### 2.2. Maximum Cumulative Angle of Traditional Scheme

In addition to dual-axis rotation of a single IMU, another line of research employs two rotation units (i.e., two rotating IMUs) to address attitude determination [44]. In this work, we focus on rotation modulation via a single IMU mounted on a dual-axis rotary mechanism. The 16-position rotation scheme [24] is the most used approach, rotation of Z + 180° is denoted as A, Z − 180° as B, Y + 180° as C, and Y − 180° as D, as illustrated in Table 1. This scheme has been widely adopted due to its established effectiveness in various applications. However, there is potential for improvement, which leads to the exploration of alternative rotation strategies.

From Equation (8), the gyro drift modulation equation is:

(11) $$C_{p}^{b}\varepsilon =\left[\begin{array}{ccc} \cos\alpha \cos\beta & -\sin\alpha \cos\beta & \sin\beta \\ \sin\alpha & \cos\alpha & 0\\ -\cos\alpha \sin\beta & \sin\alpha \sin\beta & \cos\beta \end{array}\right]\left[\begin{array}{c} \varepsilon_{x}\\ \varepsilon_{y}\\ \varepsilon_{z} \end{array}\right]$$

The attitude error of the gyro drift [41,42] is:

(12) $$\begin{array}{cc} \Phi_{\varepsilon } & =\int_{0}^{t_{r}}\left(\sum_{i=0}^{n}C_{p}^{b}\varepsilon \right)dt\\ \; & \;=\int_{0}^{t_{r}}\left(\sum_{i=0}^{n}\left[\begin{array}{c} \cos\alpha \cos\beta \varepsilon_{x}-\sin\alpha \cos\beta \varepsilon_{y}+\sin\beta \varepsilon_{z}\\ \sin\alpha \varepsilon_{x}+\cos\alpha \varepsilon_{y}\\ -\cos\alpha \sin\beta \varepsilon_{x}+\sin\alpha \sin\beta \varepsilon_{y}+\cos\beta \varepsilon_{z} \end{array}\right]\right)dt \end{array}$$

where: $t_{r}$ is the total rotation time of Sequence 1.

**Figure 2:**
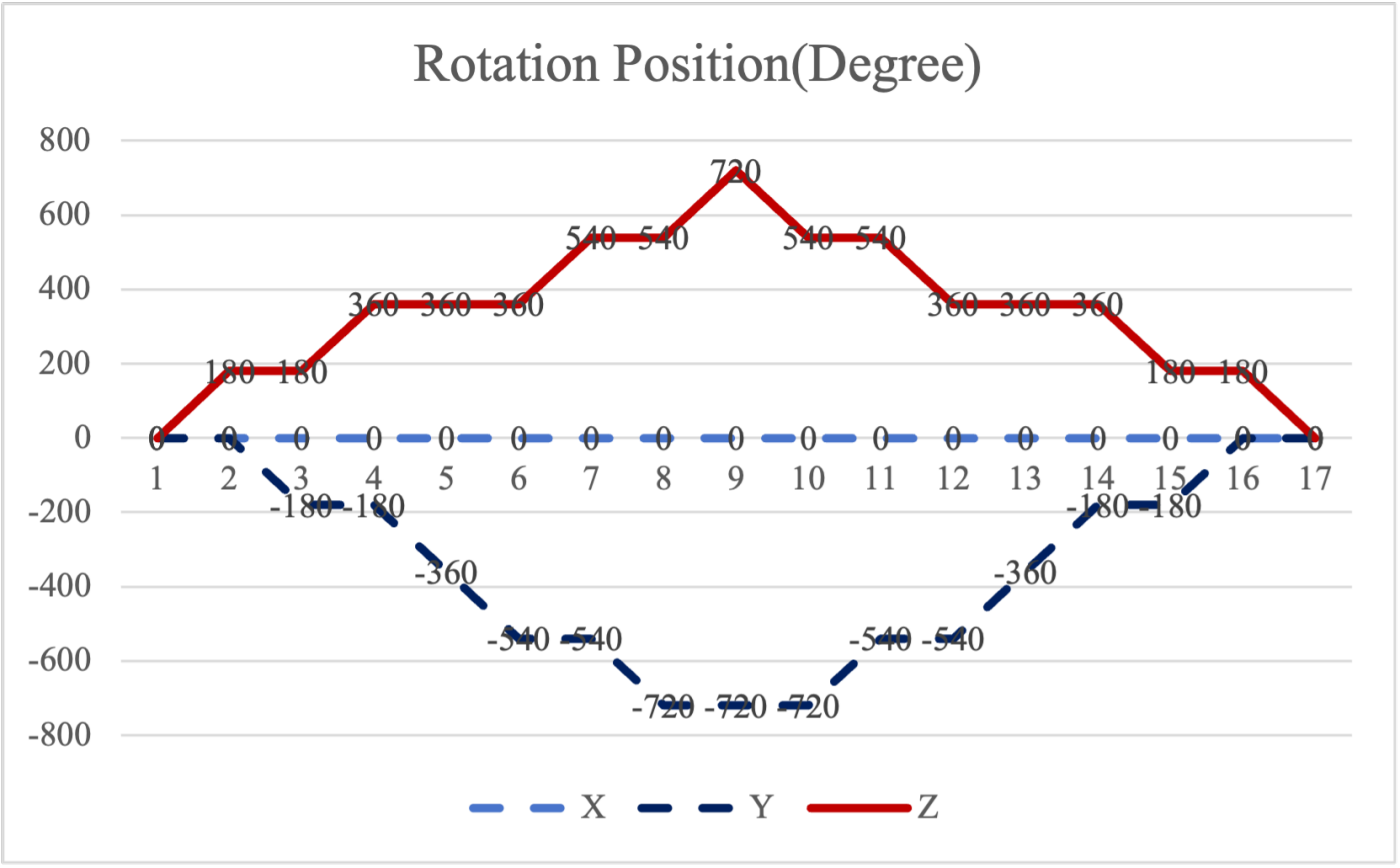
Maximum cumulative angle of 16-position scheme in [20].

Equation (12) shows that larger cumulative angles amplify the attitude residuals. The 16-position scheme suffers from a maximum cumulative rotation angle of 720° for both axes in Figure 2. This excessive rotation exacerbates the residual errors. Furthermore, the scheme cannot compensate for errors in the two rotating axes promptly within a complete cycle, leading to unmitigated inaccuracies.

### 2.3. Symmetrical Properties and Its Rotation Modulation Scheme

**Figure 3:**
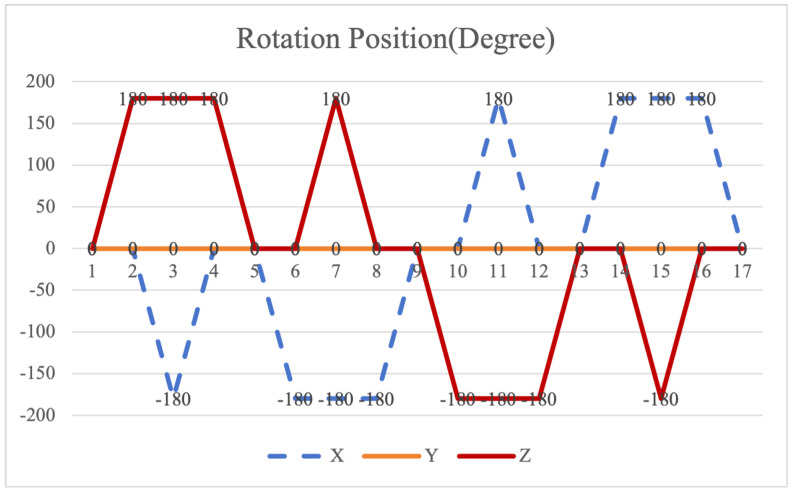
Maximumcumulative angle of 16-position scheme in [24].

**Figure 4:**
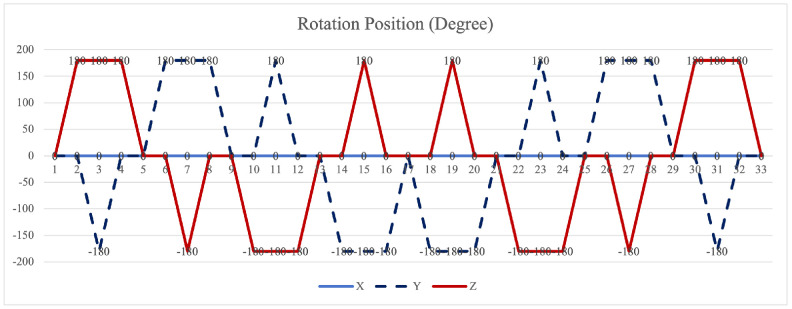
Maximum cumulative angle of 32-position scheme in [30].

The symmetrical properties of the function are crucial for eliminating errors. The rotation modulation scheme is designed to fully utilize the error propagation characteristics associated with these odd symmetrical properties. The 16-position [24] and 32-position [30] dual-axis rotational inertial navigation systems, shown in Figure 3 and Figure 4, leverage these odd symmetrical properties to create a rotational scheduling that limits the maximum cumulative rotation angle to 180°. These schemes aim to make full use of the odd symmetry of each axis and maximize the use of error propagation characteristics, which play a vital role in reducing errors. However, the influence of odd symmetry in rotation modulation schemes across multiple axes has not been adequately addressed; we propose a new 64-position rotation scheme.

## 3. Design and Methodology

To address these shortcomings, we introduce a 64-position dual-axis rotation scheme, denoting rotations of Z + 90°, Z − 90°, Y + 90°, and Y − 90° as E, F, G, and H, respectively (in Table 2). This new approach reduces the maximum cumulative rotation angle to 180°, significantly lower than the 720° in the 16-position scheme (Figure 3), thereby decreasing attitude residuals. Additionally, it enables full error compensation every 16 positions, enhancing modulation capability and improving the INS performance.

**Figure 5:**
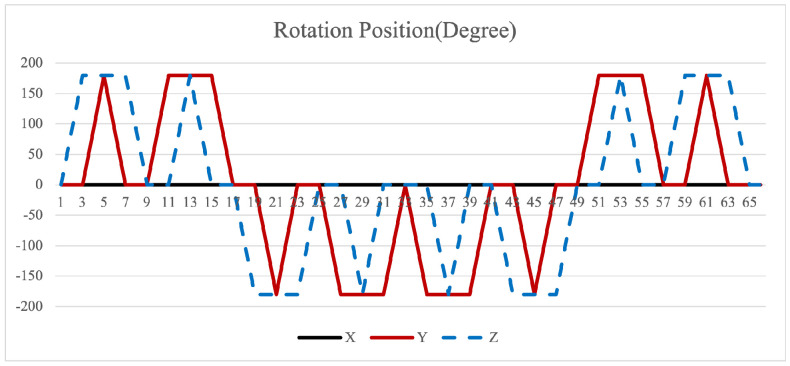
Maximum cumulative angle of 64-position scheme.

In our proposed method Figure 5, the *z*-axis positions 10 to 18 exhibit odd symmetry with respect to the *y*-axis positions 18 to 26, while *z*-axis positions 9 to 17 are odd-symmetric relative to *y*-axis positions 17 to 25. Similarly, *y*-axis positions 10 to 18 are odd-symmetric with respect to *z*-axis positions 18 to 26, and *y*-axis positions 9 to 17 correspondingly exhibit odd symmetry relative to *y*-axis positions 17 to 25. This odd-symmetric relationship appears 12 times in our approach, compared to 8 occurrences in the 16-position [24] and 4 times in the 32-position scheme [30]. Therefore, our scheme effectively leverages the odd symmetry of the rotation axis to enhance optimization.

Our rotation modulation scheme is designed to fully utilize error propagation characteristics, with the odd symmetrical properties of functions playing a crucial role in error elimination. The proposed 64-position dual-axis rotation scheme is not merely a permutation and combination of the 16 or 32-position sequence. We leveraged both the odd symmetry of the rotation order on each axis and between the two axes to modulate and reduce error propagation. Additionally, each positioning scheme features smaller rotation angles in every process, leading to reduced cumulative errors and enhanced error suppression capability along with overall performance optimization.

## 4. Attitude Error Analysis

In this analysis, we focus on a detailed comparison of the improved 64-position dual-axis rotation scheme proposed in this paper with the methodologies in [24,30]. Ref. [24] is marked as Scheme 1, ref. [30] is marked as Scheme 2, and the improved scheme proposed in this paper is Scheme 3.

### 4.1. Gyroscope Drift Error Modulation

**Figure 6:**
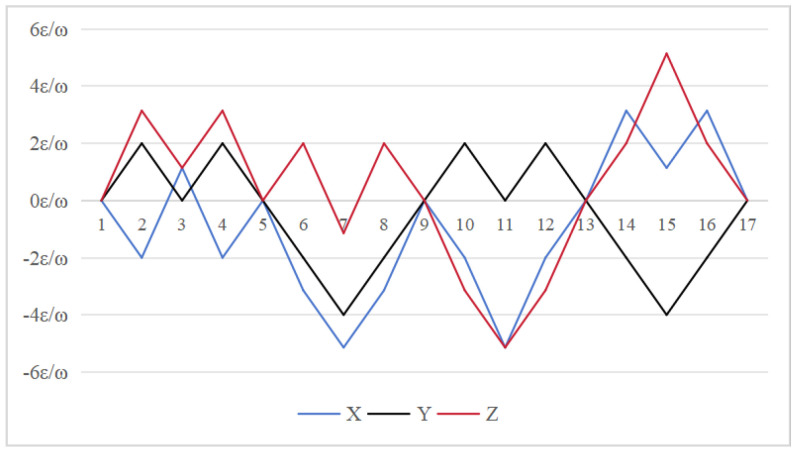
Attitude residual of Scheme 1 caused by gyroscope drift error [24].

**Figure 7:**
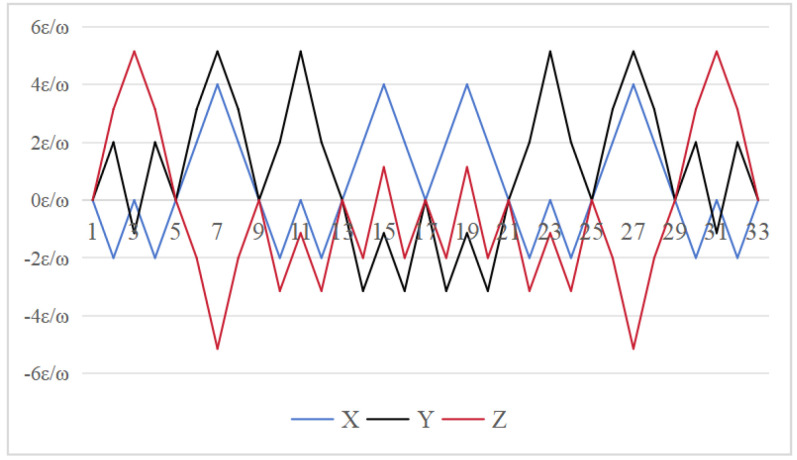
Attitude residual of Scheme 2 caused by gyroscope drift error [30].

**Figure 8:**
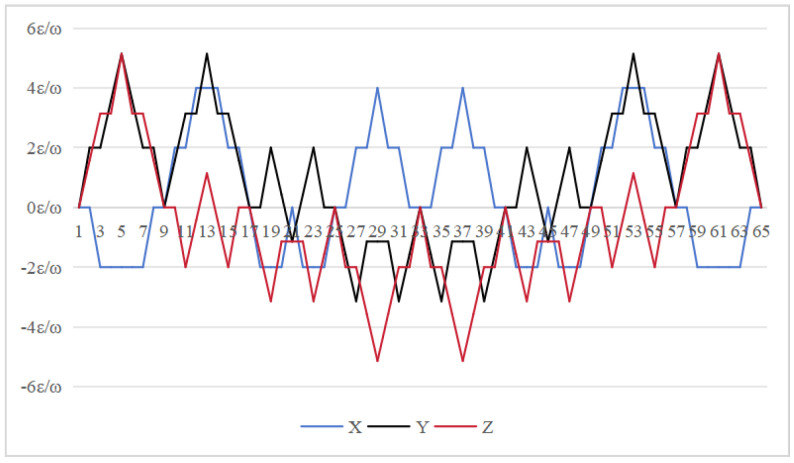
Attitude residual of Scheme 3 caused by gyroscope drift error (ours).

From Equation (12), to simplify the analysis, we assume that $\varepsilon_{x}=\varepsilon_{y}=\varepsilon_{z}=\varepsilon$. From the preceding formula, we can derive the attitude residual errors resulting from the gyro drift associated with the three schemes. The results are presented in Figure 6, Figure 7 and Figure 8, which reveal significant differences in the modulation effects of various rotation schemes on normal value errors. For the 16-position rotation scheme, the maximum accumulated errors are 4ε/ω for the *y* axis and (2+π)ε/ω for both the *x* and *z* axis. In the 32 and 64-position rotation schemes, the maximum accumulated error for the *x* axis remains at 4ε/ω, while the maximum accumulated errors for the *y* and *z* axis are reduced to (2+π)ε/ω. Both schemes have the same maximum cumulative attitude error across three axes; however, the proposed 64-position rotation schemes use a 90° rotation angle for each sequence, significantly minimizing system state changes with each rotation. This progressive modulation method effectively reduces the cumulative effect during error propagation, leading to improved error performance.The average cumulative errors for the 16-position on the *x*-axis, *y*-axis, and *z*-axis are ε/ω, 0.5ε/ω, and 0.5ε/ω, respectively. For the 32-position, they are 0.5ε/ω, ε/ω, and 0.5ε/ω, respectively. The errors for the 64-position are 0.3125ε/ω, ε/ω, and 0.5ε/ω. To maintain consistency with the duration of the 64-position sequence, the cumulative error region of the 16-position should be doubled through simple replication; this increases it to 2ε/ω, ε/ω, and ε/ω. The 64-position sequence demonstrates superior performance in terms of cumulative error in the three schemes.

### 4.2. Scale Factor Error Modulation

From Equation (8), the scale factor error modulation equation is:

(13) $$\begin{array}{cc} C_{p}^{b}S_{g}{\hat{\omega }}_{ip}^{p} & =\left[\begin{array}{ccc} \cos\alpha \cos\beta & -\sin\alpha \cos\beta & \sin\beta \\ \sin\alpha & \cos\alpha & 0\\ -\cos\alpha \sin\beta & \sin\alpha \sin\beta & \cos\beta \end{array}\right]\\ \; & \;\times \left[\begin{array}{ccc} S_{gx} & \; & \\ \; & S_{gy} & \\ \; & \; & S_{gz} \end{array}\right]\times {\hat{\omega }}_{ip}^{p}\\ \; & \;=\left[\begin{array}{ccc} S_{gx}\cos\alpha \cos\beta & -S_{gy}\sin\alpha \cos\beta & S_{gz}\sin\beta \\ S_{gx}\sin\alpha & S_{gy}\cos\alpha & 0\\ -S_{gx}\cos\alpha \sin\beta & S_{gy}\sin\alpha \sin\beta & S_{gz}\cos\beta \end{array}\right]\times {\hat{\omega }}_{ip}^{p} \end{array}$$

The attitude error resulting from the gyro scale factor error can be expressed as follows:

(14) $$\begin{array}{cc} \Phi_{S} & =\int_{0}^{t_{r}}\left(\sum_{i=0}^{n}C_{p}^{b}S_{g}{\hat{\omega }}_{ip}^{p}\right)dt\\ \; & \;=\int_{0}^{t_{r}}\sum_{i=0}^{n}\left[\begin{array}{ccc} S_{gx}\cos\alpha \cos\beta & -S_{g_{y}}\sin\alpha \cos\beta & S_{gz}\sin\beta \\ S_{gx}\sin\alpha & S_{g_{y}}\cos\alpha & 0\\ -S_{gx}\cos\alpha \sin\beta & S_{g_{y}}\sin\alpha \sin\beta & S_{gz}\cos\beta \end{array}\right]\\ \; & \;\times {\hat{\omega }}_{ip}^{p}\;dt \end{array}$$

**Figure 9:**
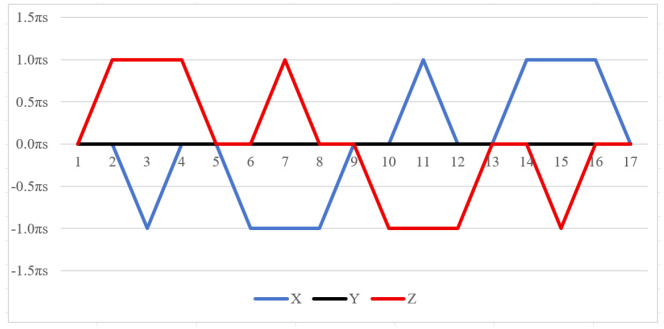
Attitude residual of Scheme 1 caused by the gyro scale factor error [24].

**Figure 10:**
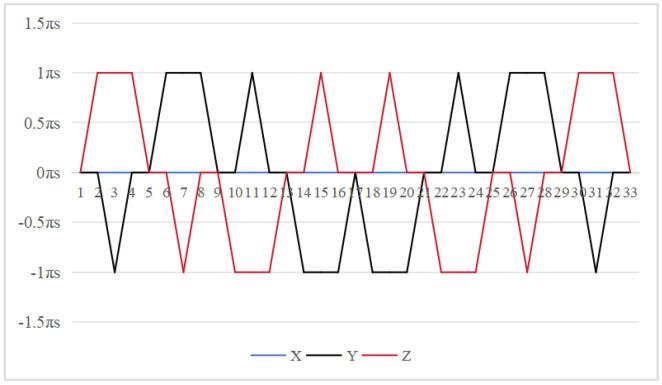
Attitude residual of Scheme 2 caused by the gyro scale factor error [30].

**Figure 11:**
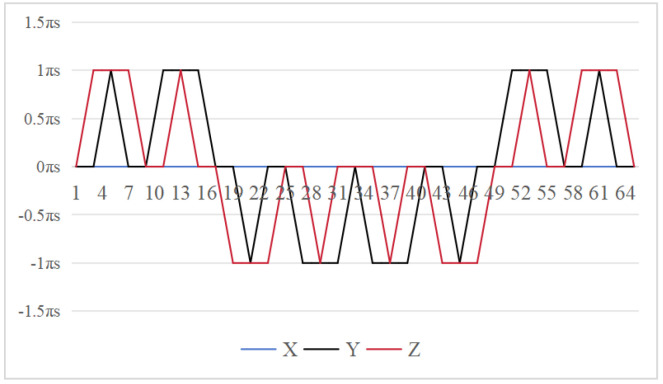
Attitude residual of Scheme 3 caused by the gyro scale factor error (ours).

To simplify the analysis, we assume that $S_{gx}=S_{gy}=S_{gz}=s$. Based on the preceding formula, we derive the attitude residual errors resulting from the gyro scale factor errors for the three schemes. The results are shown in Figure 9, Figure 10 and Figure 11, which reveal differences in the modulation effects of various rotation schemes. In the 16-position rotation scheme, the maximum accumulated attitude error on the *y* axis is 0, indicating effective performance in this direction. However, the maximum cumulative attitude errors on the *x* and *z* axes reach πs, suggesting limitations in error modulation for these axes. Although, the 32 and 64-position rotation schemes exhibit similar performance with maximum cumulative pose errors of πs for both the *y* and *z* axes. Each sequence in the 64-position scheme has an incremental change of 0.5πs, significantly improving the modulation of scale factor errors in these directions and further reducing overall error.

In summary these results suggest that the 64-position rotation scheme exhibits superior performance compared to the 16 and 32-position schemes, particularly in terms of reducing attitude errors on the *y* and *z* axes.

### 4.3. Installation Error Modulation

From Equation (8), the installation error modulation equation is:

(15) $$\begin{array}{cc} C_{p}^{b}{\left(\Delta C_{g}^{p}\right)}^{T}{\hat{\omega }}_{ip}^{p} & =\left[\begin{array}{ccc} \cos\alpha \cos\beta & -\sin\alpha \cos\beta & \sin\beta \\ \sin\alpha & \cos\alpha & 0\\ -\cos\alpha \sin\beta & \sin\alpha \sin\beta & \cos\beta \end{array}\right]\\ \; & \times \left[\begin{array}{ccc} 0 & K_{gxy} & K_{gxz}\\ K_{gyx} & 0 & K_{gyz}\\ K_{gzx} & K_{gzy} & 0 \end{array}\right]{\hat{\omega }}_{ip}^{p} \end{array}$$

The attitude error caused by the gyro installation error is:

(16) $$\begin{array}{cc} \; & \Phi_{K}=\int_{0}^{t_{r}}\left(\sum_{i=0}^{n}C_{p}^{b}{\left(\Delta C_{g}^{p}\right)}^{T}{\hat{\omega }}_{ip}^{p}\right)dt\\ \; & =\int_{0}^{t_{r}}\sum_{i=0}^{n}\left[\begin{array}{ccc} \cos\alpha \cos\beta & -\sin\alpha \cos\beta & \sin\beta \\ \sin\alpha & \cos\alpha & 0\\ -\cos\alpha \sin\beta & \sin\alpha \sin\beta & \cos\beta \end{array}\right]\\ \; & \times \left[\begin{array}{ccc} 0 & K_{gxy} & K_{gxz}\\ K_{gyx} & 0 & K_{gyz}\\ K_{gzx} & K_{gzy} & 0 \end{array}\right]{\hat{\omega }}_{ip}^{p}dt \end{array}$$

According to Equation (16), the rotational angle is a key factor influencing installation error modulation effectiveness. The larger cumulative rotation angle in the 16-position scheme diminishes modulation effectiveness, resulting in higher attitude errors. Conversely, the 32-position and 64-position schemes, with their smaller cumulative rotation angles, exhibit reduced attitude errors compared to the 16-position scheme. The cumulative rotation angle is crucial for minimizing the impact of installation errors and enhancing overall system performance.

To simplify the analysis, let $\;K_{gxy}=\;K_{gxz}=\;K_{gyx}=\;K_{gyz}=\;K_{gzx}=\;K_{gzy}=k$. From the preceding formula, we can derive the attitude residual errors of the gyro installation errors in the three schemes.

**Figure 12:**
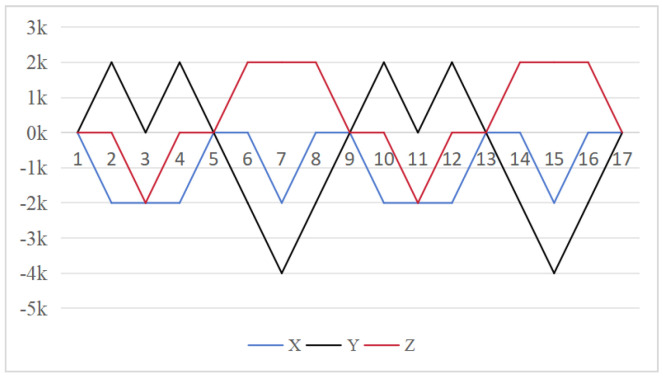
Attitude residual of Scheme 1 caused by installation error [24].

**Figure 13:**
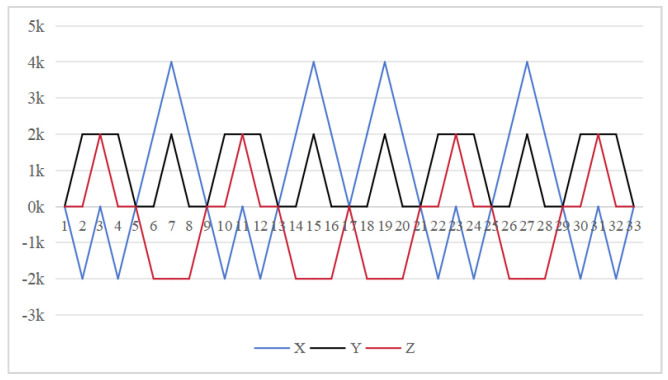
Attitude residual of Scheme 2 caused by installation error [30].

**Figure 14:**
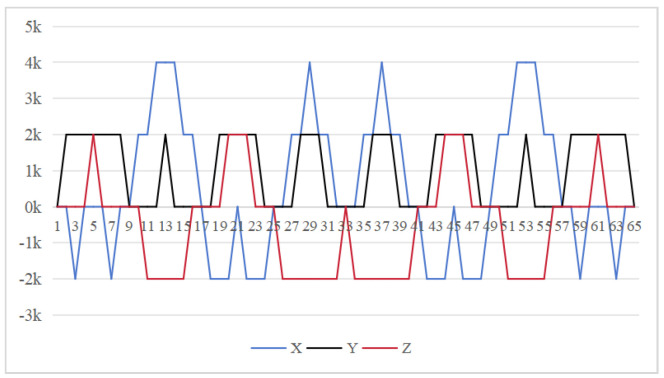
Attitude residual of Scheme 3 caused by installation error (ours).

Figure 12, Figure 13 and Figure 14 demonstrate how installation errors affect the remaining attitude error. For the 16-position rotation scheme, the maximum cumulative errors of the *x* and *z* axes are both 2k, while the maximum cumulative error of the *y* axis is 4k. In contrast, the 32-position and 64-position schemes both maintain a maximum cumulative error of 4k for the *x* axis and a maximum error of 2k for the *y* and *z* axes.This suggests that they have a similar impact on installation factor errors.

### 4.4. Summary of Attitude Errors Caused by Gyro Drift, Scale Factor Error and Installation Error

The modulation effect is related to the rotation angles $\alpha =\omega_{z}*t$ and $\beta =\omega_{y}*t$. The maximum cumulative rotation angle for the 16-position scheme [20] reaches 720°, whereas the 16-position scheme [24], the 32-position scheme [30], and the 64-position scheme limit this angle to 180°. This significant difference means that the errors in the *y* and *z* axes of the 16-position scheme [20] cannot effectively cancel out, leading to larger attitude error residuals. The maximum cumulative rotation angle of the 16-position scheme [24] reaches 180°, as do those for both the 32-position scheme [30] and the 64-position scheme. Although there is no numerical difference of maximum cumulative attitude residual among these three schemes regarding constant error, scale factor error, and installation error. Both the 32- and 64-position schemes exhibit similar maximum values concerning these errors. However, each schedule of the 64-position scheme involves smaller rotation angles, leading to reduced cumulative errors and demonstrating better error suppression capabilities along with overall performance optimization. Additionally, the average cumulative errors from the 64-position schemes indicate superior performance due to gyroscope bias.

## 5. Velocity Error Analysis

In this section, we conduct a comparison and analysis of the improved 64-position dual-axis rotation scheme. For simplifying our analysis, we assume that the carrier remains static. Under this assumption, we can derive the error transmission equation for the RINS.

(17) $$\delta \;v=\int_{0}^{t_{r}}\sum_{i=0}^{n}\left(f_{n}\times \phi \right)dt=\int_{0}^{t_{r}}\sum_{i=0}^{n}\left(\left[\begin{array}{c} 0\\ 0\\ g \end{array}\right]\times \left[\begin{array}{c} \phi_{E}\\ \phi_{N}\\ \phi_{U} \end{array}\right]\right)dt$$

We focus exclusively on the scale factor error and installation error when discussing velocity errors, as the attitude error resulting from the regular value error in the three schemes is 0. The eastward velocity error $\delta v_{E}$ and the northward velocity error $\delta v_{N}$ for each of the three schemes are presented in Table 3, Table 4 and Table 5.

The 64-position scheme demonstrates superior performance in modulating velocity errors, exhibiting higher accuracy and stability compared to both the 16-position [24] and 32-position schemes [30]. The modulation effects of the three rotation schemes on velocity errors show that the average cumulative eastward velocity error for the 16-position scheme [24] is $-1.5kgt_{r}$, while the northward velocity error is $\left(-\pi s-10k\right)gt_{r}$. For the 32-position scheme, the average cumulative eastward velocity error is $-\left(0\pi s+17.75k\right)gt_{r}$, and the northward velocity error is $8.5kgt_{r}$. In contrast, the 64-position scheme exhibits an average cumulative eastward velocity error of $-\left(0\pi s+33k\right)gt_{r}$, and a northward velocity error of $16.5kgt_{r}$. During the rotation modulation process, the scale factor error significantly influences the system’s performance. Notably, both the 32 and 64-position schemes achieve complete modulation of the scale factor error, resulting in superior modulation effects compared to the 16-position scheme. Due to the unique symmetry in the rotation sequence designed to maximize benefits on each axis and between both axes, the rotation period $t_{r}$ for the 32-position scheme is twice that of the 64-position scheme, resulting in a smaller average cumulative error for the latter. Additionally, the smaller adjustments made by each sequential rotation in the 64-position scheme further reduce the final error.

Overall, the 64-position scheme demonstrates superior performance in modulating velocity errors, exhibiting higher accuracy and stability compared to both the 16-position and 32-position schemes.

## 6. Simulation Results and Analysis

The simulation conditions are defined: the gyro scale factor error is $1\times 10^{-5}$, the drift is $0.01^{\circ }/h$, the installation error is $10^{\prime \prime }$, the acceleration scale factor error is $5\times 10^{-5}$, the bias is 50 µg, the installation error is $10^{\prime \prime }$ and the geographical coordinates of the carrier are $26.502^{\circ }$ north latitude and $106.691^{\circ }$ east longitude. The initial position, attitude and velocity errors are all set to 0.The uniform rotation speed ω is set as $16^{\circ }/s$, the angular acceleration *a* as $8^{\circ }/s^{2}$. The first scheme labelled Option 1 is the traditional 16-position rotation scheme in [20], and the second scheme labelled Option 2 is the improved 16-position rotation scheme proposed in [24]. The third scheme labelled Option 3 the improved 16-position rotation scheme proposed in [25], the fourth scheme labelled Option 4 is the 32-position rotation scheme proposed in [30], and the fifth scheme labelled Option 5 (ours) is the 64-position rotation scheme enhanced in this paper. The analysis of the attitude error and velocity error of the additional scheme is the same as the above scheme. The effects of various rotation schemes on system navigation are shown in Figure 13.

The navigation error selection principle under different schemes [25] is:

(18) $$x_{max}=\max\left|x\right|$$

where *x* is the error term of the inertial navigation system for different schemes, specifically the latitude error δL, longitude error δλ, eastward velocity error $\delta v_{E}$, northward velocity error $\delta v_{N}$, roll angle error Δγ, pitch angle error Δθ, and yaw angle error Δψ. The error values are shown in Table 6.

The simulation data indicate that Option 5 exhibits substantial optimization effects across all error indicators, particularly in latitude error, longitude error, eastward velocity error and heading angle error. Compared to other schemes, Option 5 consistently achieves the lowest error values, underscoring its effectiveness in error suppression.

To verify the effectiveness of the simulation experiment, we conducted 50 Monte Carlo simulations. Specifically, when compared with Option 1, Option 5 reduces latitude error by 68.3% and longitude error by 62.8%. Additionally, it decreases eastward velocity error and northward velocity error by 71.8% and 71.3%, respectively. Other error indicators also show significant reductions, demonstrating marked improvement. In comparison to Option 2, Option 5 achieves over a 30% reduction in longitude error, eastward velocity error, and yaw error, further reinforcing its adaptability and robustness in complex error scenarios. When evaluated against Options 3 and 4, Option 5 demonstrates significant optimization across nearly all error indicators, with roll and pitch errors reduced by 42.9% and 43.3%, respectively, leading to a substantial overall decrease in error levels. The results show that the improved scheme enhances all error metrics greatly compared to existing rotation modulation schemes.

## 7. Experimental Verification

**Figure 15:**
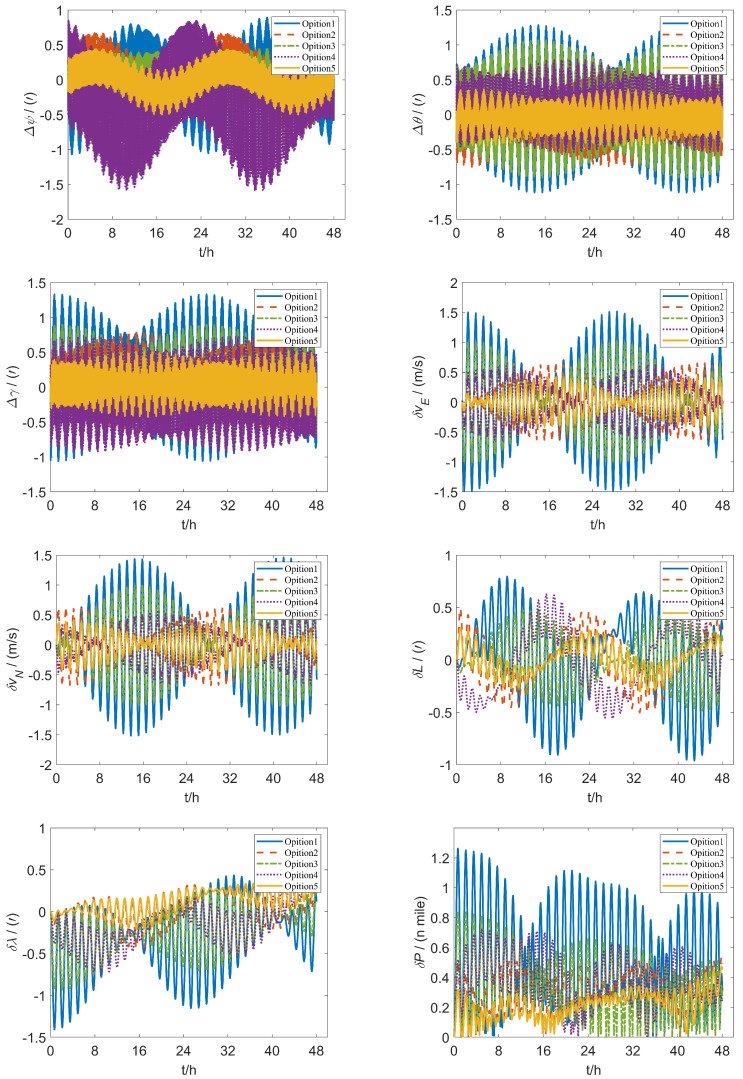
Effect of different schemes on system navigation.

To further validate the superiority of the modified rotation scheme, rotation experiments were conducted on the five schemes. Initial alignment is essential for INS accuracy. Previous studies improved alignment by optimizing data utilization [39,45,46] and compensating for lever arm effects [40,47]. Therefore, this experiment performed a 15-min IMU initial alignment before rotation modulation to ensure accuracy. Figure 14 shows the experimental setup for rotation modulation, including a fiber optic IMU, a dual-axis turntable and a computer. The rotation schemes are programmed using QT, with instructions transmitted to the dual-axis turntable via the computer. Subsequently, data from the rotated and modulated IMU is relayed to the computer through the RS422 serial port. The IMU is equipped with three quartz accelerators and three fiber optic gyroscopes with an accuracy of 50 µg and $0.03^{\circ }/h$. The location of the initial position is $116.2248^{\circ }$ E and $40.2448^{\circ }$ N. The initial velocity is zero, the laboratory temperature was 20 °C and the humidity was 30%.The turntable system is bifurcated into an outer frame and an inner frame, offering angular positioning accuracy of ±1.0", while the solution for the inner and outer axis speed resolution is $0.0001^{\circ }/s$. The parameters for the enhanced dual-axis rotation experiment are as follows: the dual-axis turntable rotation angular speed ω is set as $16^{\circ }/s$, the rotation acceleration *a* as $8^{\circ }/s^{2}$. Figure 15 illustrates the experimental results of five dual-axis rotation schemes over a duration of 24 h. The error values are shown in Table 7.

**Figure 16:**
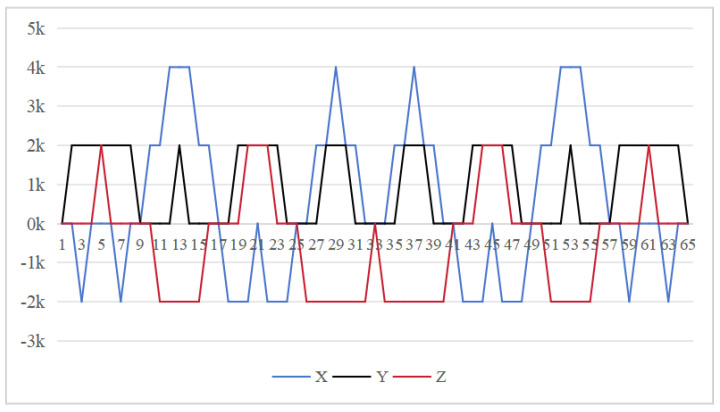
Physics experimental platforms and devices.

**Figure 17:**
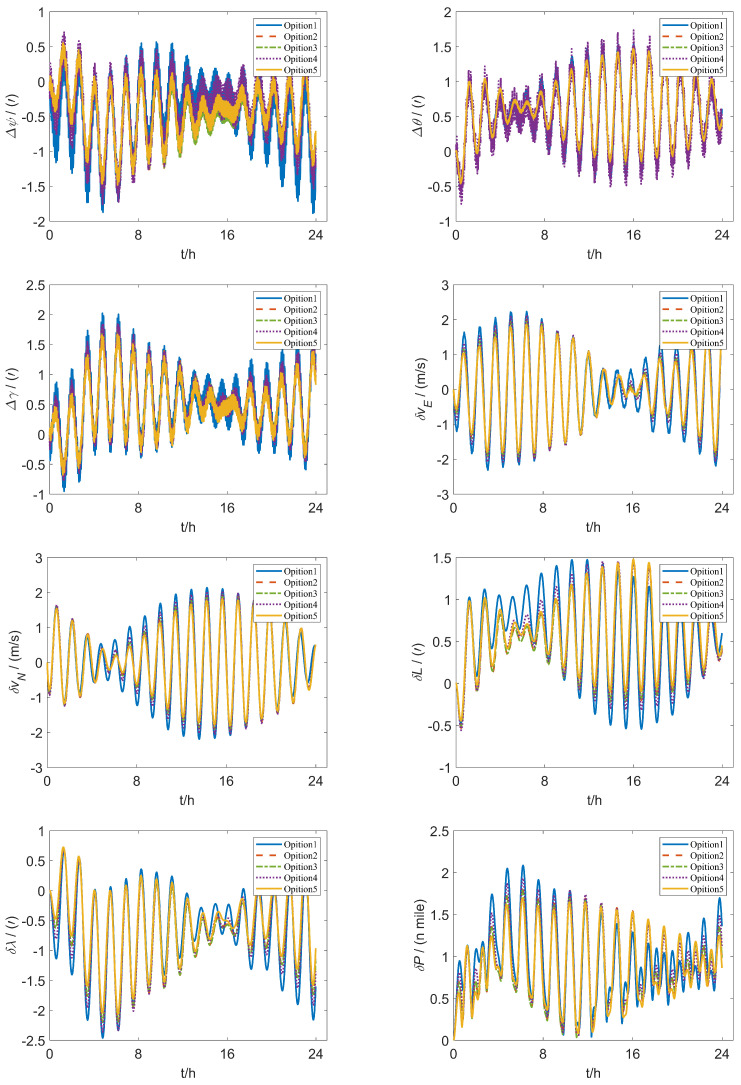
Error impact of five schemes over a 24-h duration.

Figure 16 shows the experimental setup for rotation modulation, including a fiber optic IMU, a dual-axis turntable and a computer. The rotation schemes are programmed using QT, with instructions transmitted to the dual-axis turntable via the computer. Subsequently, data from the rotated and modulated IMU is relayed to the computer through the RS422 serial port. The IMU is equipped with three quartz accelerators and three fiber optic gyroscopes with an accuracy of 50 µg and $0.03^{\circ }/h$. The location of the initial position is $116.2248^{\circ }$ E and $40.2448^{\circ }$ N. The initial velocity is zero, the laboratory temperature was 20 °C and the humidity was 30%. The turntable system is bifurcated into an outer frame and an inner frame, offering angular position positioning accuracy of $\pm 1.0^{\prime \prime }$, while the solution for the inner and outer axis speed resolution is 0.0001 °/*s*. The parameters for the enhanced dual-axis rotation experiment are as follows: the dual-axis turntable rotation angular speed ω is set as $16^{\circ }/s$, the rotation acceleration *a* as $8^{\circ }/s^{2}$. Figure 17 illustrates the experimental results of five dual-axis rotation schemes over a duration of 24 h. The error values are shown in Table 7.

The experimental data reveal that Option 5 demonstrates substantial optimization effects across most error indicators, particularly in longitude error, eastward velocity error, northward velocity error and yaw angle error. When compared to other schemes, Option 5 achieves the lowest overall error values, indicating superior error suppression capabilities and enhanced system stability.

Specifically, relative to Option 1, Option 5 reduces longitude error by 15.3%, eastward velocity error by 19.7% northward velocity error by 17.1%, and yaw angle error by 21.5%. In comparison to the optimized Option 2, it reduces longitude error by 6.9%, eastward velocity error by 7.8%, northward velocity error by 6.4% and yaw error by 7.0%. When contrasted with Option 3, Option 5 achieves reductions of 6.3% in eastward velocity error and 5.6% in yaw error, further enhancing overall navigation accuracy. Additionally, when compared to Option 4, Option 5 reduces longitude error, eastward velocity error and northward velocity error by 11.6%, 15.3%, 14.5%, respectively, while also reducing yaw error by 9.0%. This demonstrates a greater capacity for error compensation.

The experimental results indicate that Option 5 achieved a lower navigation error compared to the other options. This demonstrates its advantage in improving navigation performance, providing a reference for the design of precise rotational inertial navigation systems under complex error conditions.

## 8. Conclusions

To address the limitations of existing rotation schemes (e.g., the 16-position rotation scheme) in inertial device error modulation—specifically, the excessive cumulative angles of the rotating axis during rotation modulation and the inability to promptly compensate for IMU errors—this study proposes an optimal 64-position rotation scheme. The core design of this scheme incorporates odd symmetry in the rotation sequence, which is intended to maximize the error suppression benefits on every single axis and across multiple axes, thereby effectively adjusting and minimizing IMU error propagation.

To verify the effectiveness of the proposed 64-position rotation scheme, this study systematically investigates the propagation characteristics of critical errors in inertial devices, including gyro drift, scale factor errors, and installation errors. Quantitative and qualitative analyses are performed to clarify the influence mechanisms of these errors on the residual attitude and velocity errors induced during the rotation modulation process, which provides a theoretical basis for the performance evaluation of the proposed scheme.

Simulation and experimental results show that the proposed 64-position rotation scheme outperforms the existing schemes in [24,30] in attitude and velocity error suppression, with superior accuracy and reliability. Specifically, it optimizes all error evaluation metrics, reduces latitude, longitude, eastward velocity, and yaw errors significantly, and exhibits enhanced adaptability and robustness under complex error scenarios.

Furthermore, experimental results validate the superiority of the improved scheme in enhancing navigation accuracy and system stability. This innovative approach not only provides a valuable perspective for optimizing inertial navigation systems but also holds promise for practical applications. Currently, the research is primarily based on simulations and laboratory tests. Future work will focus on validating the performance of the improved scheme correlated-noise models in correlated-noise models, dynamic environments and its application in the initial alignment process. Plans include utilizing a smaller, more integrated turntable device to conduct tests in real navigation scenarios, thereby evaluating the effectiveness of the scheme in dynamic errors.

## Figures and Tables

**Table 1:** The Scheme of 16-position dual-axis rotation.

Position	Axis and Angle	Position	Axis and Angle
1	A	9	B
2	C	10	D
3	B	11	A
4	D	12	C
5	D	13	C
6	B	14	A
7	C	15	D
8	A	16	B

**Table 2:** The Scheme of 64-position dual-axis rotation.

Group 1		Group 2		Group 3		Group 4	
**Position**	**Axis**	**Position**	**Axis**	**Position**	**Axis**	**Position**	**Axis**
1	E	17	F	33	H	49	G
2	E	18	F	34	H	50	G
3	H	19	G	35	E	51	F
4	H	20	G	36	E	52	F
5	G	21	H	37	F	53	E
6	G	22	H	38	F	54	E
7	F	23	E	39	G	55	H
8	F	24	E	40	G	56	H
9	G	25	H	41	F	57	E
10	G	26	H	42	F	58	E
11	F	27	E	43	G	59	H
12	F	28	E	44	G	60	H
13	E	29	F	45	H	61	G
14	E	30	F	46	H	62	G
15	H	31	G	47	E	63	F
16	H	32	G	48	E	64	F

**Table 3:** Velocity residual error of Scheme 1.

Position	$\delta v_{E}\left(m/s\right)$	$\delta v_{N}\left(m/s\right)$	Position	$\delta v_{E}\left(m/s\right)$	$\delta v_{N}\left(m/s\right)$
1	$-2kgt_{r}$	$-2kgt_{r}$	5	$-2kgt_{r}$	$-6kgt_{r}$
2	$-2kgt_{r}$	$\left(\pi s-4k\right)gt_{r}$	6	$2kgt_{r}$	$\left(-\pi s-8k\right)gt_{r}$
3	$-4kgt_{r}$	$\left(\pi s-6k\right)gt_{r}$	7	$4kgt_{r}$	$\left(-2\pi s-8k\right)gt_{r}$
4	$-4kgt_{r}$	$\left(\pi s-6k\right)gt_{r}$	8	$4kgt_{r}$	$\left(-2\pi s-8k\right)gt_{r}$
9	$2kgt_{r}$	$\left(-2\pi s-10k\right)gt_{r}$	13	$2kgt_{r}$	$\left(-2\pi s-14k\right)gt_{r}$
10	$2kgt_{r}$	$\left(-3\pi s-12k\right)gt_{r}$	14	$6kgt_{r}$	$\left(-\pi s-16k\right)gt_{r}$
11	0	$\left(-3\pi s-14k\right)gt_{r}$	15	$8kgt_{r}$	$-16kgt_{r}$
12	0	$\left(-3\pi s-14k\right)gt_{r}$	16	$8kgt_{r}$	$-16kgt_{r}$

**Table 4:** Velocity residual error of Scheme 2.

Position	$\delta v_{E}\left(m/s\right)$	$\delta v_{N}\left(m/s\right)$	Position	$\delta v_{E}\left(m/s\right)$	$\delta v_{N}\left(m/s\right)$
1	$-2kgt_{r}$	$-2kgt_{r}$	9	$-\left(-2\pi s+10k\right)gt_{r}$	$2kgt_{r}$
2	$-\left(\pi s+4k\right)gt_{r}$	$-2kgt_{r}$	10	$-\left(-3\pi s+12k\right)gt_{r}$	$2kgt_{r}$
3	$-\left(\pi s+6k\right)gt_{r}$	$-4kgt_{r}$	11	$-\left(-3\pi s+14k\right)gt_{r}$	0
4	$-\left(\pi s+6k\right)gt_{r}$	$-4kgt_{r}$	12	$-\left(-3\pi s+14k\right)gt_{r}$	0
5	$-6kgt_{r}$	$-2kgt_{r}$	13	$-\left(-2\pi s+14k\right)gt_{r}$	$2kgt_{r}$
6	$-\left(-\pi s+8k\right)gt_{r}$	$2kgt_{r}$	14	$-\left(-\pi s+16k\right)gt_{r}$	$6kgt_{r}$
7	$-\left(-2\pi s+8k\right)gt_{r}$	$4kgt_{r}$	15	$-16kgt_{r}$	$8kgt_{r}$
8	$-\left(-2\pi s+8k\right)gt_{r}$	$4kgt_{r}$	16	$-16kgt_{r}$	$8kgt_{r}$
17	$-\left(\pi s+16k\right)gt_{r}$	$10kgt_{r}$	25	$-\left(\pi s+26k\right)gt_{r}$	$14kgt_{r}$
18	$-\left(2\pi s+18k\right)gt_{r}$	$14kgt_{r}$	26	$-28kgt_{r}$	$18kgt_{r}$
19	$-\left(3\pi s+18k\right)gt_{r}$	$16kgt_{r}$	27	$-\left(-\pi s+28k\right)gt_{r}$	$20kgt_{r}$
20	$-\left(3\pi s+18k\right)gt_{r}$	$16kgt_{r}$	28	$-\left(-\pi s+28k\right)gt_{r}$	$20kgt_{r}$
21	$-\left(3\pi s+22k\right)gt_{r}$	$14kgt_{r}$	29	$-\left(-\pi s+30k\right)gt_{r}$	$18kgt_{r}$
22	$-\left(2\pi s+24k\right)gt_{r}$	$14kgt_{r}$	30	$-32kgt_{r}$	$18kgt_{r}$
23	$-\left(2\pi s+26k\right)gt_{r}$	$12kgt_{r}$	31	$-34kgt_{r}$	$16kgt_{r}$
24	$-\left(2\pi s+26k\right)gt_{r}$	$12kgt_{r}$	32	$-34kgt_{r}$	$16kgt_{r}$

**Table 5:** Velocity residual error of Scheme 3.

Position	$\delta v_{E}\left(m/s\right)$	$\delta v_{N}\left(m/s\right)$	Position	$\delta v_{E}\left(m/s\right)$	$\delta v_{N}\left(m/s\right)$
1	$-2kgt_{r}$	0	9	$-\left(2.5\pi s+14k\right)gt_{r}$	$-2kgt_{r}$
2	$-4kgt_{r}$	$-2kgt_{r}$	10	$-\left(3.5\pi s+14k\right)gt_{r}$	0
3	$-\left(0.5\pi s+6k\right)gt_{r}$	$-2kgt_{r}$	11	$-\left(4.5\pi s+14k\right)gt_{r}$	$4kgt_{r}$
4	$-\left(1.5\pi s+8k\right)gt_{r}$	$-2kgt_{r}$	12	$-\left(5.5\pi s+16k\right)gt_{r}$	$8kgt_{r}$
5	$-\left(2\pi s+10k\right)gt_{r}$	$-2kgt_{r}$	13	$-\left(6.5\pi s+16k\right)gt_{r}$	$12kgt_{r}$
6	$-\left(2\pi s+12k\right)gt_{r}$	$-4kgt_{r}$	14	$-\left(7.5\pi s+16k\right)gt_{r}$	$14kgt_{r}$
7	$-\left(2\pi s+14k\right)gt_{r}$	$-4kgt_{r}$	15	$-\left(8\pi s+16k\right)gt_{r}$	$16kgt_{r}$
8	$-\left(2\pi s+14k\right)gt_{r}$	$-4kgt_{r}$	16	$-\left(8\pi s+16k\right)gt_{r}$	$16kgt_{r}$
17	$-\left(8\pi s+16k\right)gt_{r}$	$14kgt_{r}$	25	$-\left(5.5\pi s+26k\right)gt_{r}$	$4kgt_{r}$
18	$-\left(8\pi s+18k\right)gt_{r}$	$12kgt_{r}$	26	$-\left(4.5\pi s+26k\right)gt_{r}$	$6kgt_{r}$
19	$-\left(7.5\pi s+20k\right)gt_{r}$	$10kgt_{r}$	27	$-\left(3.5\pi s+28k\right)gt_{r}$	$8kgt_{r}$
20	$-\left(6.5\pi s+22k\right)gt_{r}$	$10kgt_{r}$	28	$-\left(2.5\pi s+30k\right)gt_{r}$	$12kgt_{r}$
21	$-\left(6\pi s+24k\right)gt_{r}$	$8kgt_{r}$	29	$-\left(1.5\pi s+32k\right)gt_{r}$	$14kgt_{r}$
22	$-\left(6\pi s+26k\right)gt_{r}$	$6kgt_{r}$	30	$-\left(0.5\pi s+32k\right)gt_{r}$	$16kgt_{r}$
23	$-\left(6\pi s+26k\right)gt_{r}$	$4kgt_{r}$	31	$-32kgt_{r}$	$16kgt_{r}$
24	$-\left(6\pi s+26k\right)gt_{r}$	$4kgt_{r}$	32	$-32kgt_{r}$	$16kgt_{r}$
33	$-\left(-0.5\pi s+32k\right)gt_{r}$	$16kgt_{r}$	41	$-\left(-6\pi s+38k\right)gt_{r}$	$26kgt_{r}$
34	$-\left(-1.5\pi s+32k\right)gt_{r}$	$18kgt_{r}$	42	$-\left(-6\pi s+40k\right)gt_{r}$	$24kgt_{r}$
35	$-\left(-2.5\pi s+34k\right)gt_{r}$	$20kgt_{r}$	43	$-\left(-6.5\pi s+42k\right)gt_{r}$	$22kgt_{r}$
36	$-\left(-3.5\pi s+36k\right)gt_{r}$	$24kgt_{r}$	44	$-\left(-7.5\pi s+44k\right)gt_{r}$	$22kgt_{r}$
37	$-\left(-4.5\pi s+38k\right)gt_{r}$	$26kgt_{r}$	45	$-\left(-8\pi s+46k\right)gt_{r}$	$20kgt_{r}$
38	$-\left(-5.5\pi s+38k\right)gt_{r}$	$28kgt_{r}$	46	$-\left(-8\pi s+48k\right)gt_{r}$	$18kgt_{r}$
39	$-\left(-6\pi s+38k\right)gt_{r}$	$28kgt_{r}$	47	$-\left(-8\pi s+48k\right)gt_{r}$	$16kgt_{r}$
40	$-\left(-6\pi s+38k\right)gt_{r}$	$28kgt_{r}$	48	$-\left(-8\pi s+48k\right)gt_{r}$	$16kgt_{r}$
49	$-\left(-7.5\pi s+48k\right)gt_{r}$	$18kgt_{r}$	57	$-\left(-2\pi s+52k\right)gt_{r}$	$36kgt_{r}$
50	$-\left(-6.5\pi s+48k\right)gt_{r}$	$20kgt_{r}$	58	$-\left(-2\pi s+54k\right)gt_{r}$	$34kgt_{r}$
51	$-\left(-5.5\pi s+48k\right)gt_{r}$	$24kgt_{r}$	59	$-\left(-1.5\pi s+56k\right)gt_{r}$	$34kgt_{r}$
52	$-\left(-4.5\pi s+50k\right)gt_{r}$	$28kgt_{r}$	60	$-\left(-0.5\pi s+58k\right)gt_{r}$	$34kgt_{r}$
53	$-\left(-3.5\pi s+50k\right)gt_{r}$	$32kgt_{r}$	61	$-60kgt_{r}$	$34kgt_{r}$
54	$-\left(-2.5\pi s+50k\right)gt_{r}$	$34kgt_{r}$	62	$-62kgt_{r}$	$32kgt_{r}$
55	$-\left(-2\pi s+50k\right)gt_{r}$	$36kgt_{r}$	63	$-64kgt_{r}$	$32kgt_{r}$
56	$-\left(-2\pi s+50k\right)gt_{r}$	$36kgt_{r}$	64	$-64kgt_{r}$	$32kgt_{r}$

**Table 6:** 48 h navigation error under different schemes.

Scheme	$\delta L{(}^{\prime })$	$\delta \lambda {(}^{\prime })$	$\delta v_{E}\left(m/s\right)$	$\delta v_{N}\left(m/s\right)$	$\Delta \gamma {(}^{\prime })$	$\Delta \theta {(}^{\prime })$	$\Delta \psi {(}^{\prime })$
Option 1 [20]	0.966	1.409	1.516	1.513	1.334	1.283	1.069
Option 2 [24]	0.502	0.584	0.652	0.678	0.785	0.751	0.773
Option 3 [25]	0.483	0.933	1.023	1.015	0.900	1.071	0.585
Option 4 [30]	0.626	0.730	0.635	0.645	0.933	0.794	1.568
Option 5 (Ours)	0.306	0.524	0.427	0.434	0.513	0.449	0.499
Option 5 vs. Option 1	68.3%	62.8%	71.8%	71.3%	61.5%	65.0%	53.3%
Option 5 vs. Option 2	39.0%	10.3%	34.5%	36.0%	34.6%	40.2%	35.4%
Option 5 vs. Option 3	36.6%	43.8%	58.3%	57.2%	43.0%	58.1%	14.7%
Option 5 vs. Option 4	51.1%	28.2%	32.8%	32.7%	45.0%	43.5%	68.5%

**Table 7:** Twenty-four hour navigation error under different schemes.

Scheme	$\delta L{(}^{\prime })$	$\delta \lambda {(}^{\prime })$	$\delta v_{E}\left(m/s\right)$	$\delta v_{N}\left(m/s\right)$	$\Delta \gamma {(}^{\prime })$	$\Delta \theta {(}^{\prime })$	$\Delta \psi {(}^{\prime })$
Option 1 [20]	1.472	2.428	2.309	2.191	2.022	1.534	1.885
Option 2 [24]	1.480	2.208	2.009	1.942	1.817	1.457	1.591
Option 3 [25]	1.451	2.209	1.978	1.931	1.791	1.462	1.566
Option 4 [30]	1.407	2.326	2.189	2.126	1.828	1.463	1.626
Option 5 (Ours)	1.475	2.056	1.853	1.817	1.667	1.471	1.479
Option 5 vs. Option 1	-	15.3%	19.7%	17.1%	17.6%	4.1%	21.5%
Option 5 vs. Option 2	0.3%	6.9%	7.8%	6.4%	8.3%	-	7.0%
Option 5 vs. Option 3	-	6.9%	6.3%	5.9%	6.9%	-	5.6%
Option 5 vs. Option 4	-	11.6%	15.3%	14.5%	8.8%	-	9.0%

## Data Availability

No new data were created or analyzed in this study. Data sharing is not applicable to this article.
